# Effect of obesity on the associations of 25-hydroxyvitamin D with prevalent and incident distal sensorimotor polyneuropathy: population-based KORA F4/FF4 study

**DOI:** 10.1038/s41366-022-01122-2

**Published:** 2022-04-26

**Authors:** Haifa Maalmi, Christian Herder, Cornelia Huth, Wolfgang Rathmann, Gidon J. Bönhof, Margit Heier, Wolfgang Koenig, Michael Roden, Annette Peters, Dan Ziegler, Barbara Thorand

**Affiliations:** 1 Institute for Clinical Diabetology, German Diabetes Center, Leibniz Center for Diabetes Research at Heinrich-Heine-University Düsseldorf, Düsseldorf, Germany; 2 German Center for Diabetes Research, Partner Düsseldorf, München-Neuherberg, Germany; 3 Department of Endocrinology and Diabetology, Medical Faculty and University Hospital Düsseldorf, Heinrich-Heine-University Düsseldorf, Düsseldorf, Germany; 4 Institute of Epidemiology, Helmholtz Zentrum München, German Research Center for Environmental Health, Neuherberg, Germany; 5 German Center for Diabetes Research, Partner Neuherberg, München-Neuherberg, Germany; 6 Institute for Biometrics and Epidemiology, German Diabetes Center, Leibniz Center for Diabetes Research at Heinrich-Heine-University Düsseldorf, Düsseldorf, Germany; 7 KORA Study Centre, University Hospital of Augsburg, Augsburg, Germany; 8 Institute of Epidemiology and Medical Biometry, University of Ulm, Ulm, Germany; 9 Deutsches Herzzentrum München, Technische Universität München, München, Germany; 10 German Centre for Cardiovascular Research (DZHK e.V.), Partner Site München Heart Alliance, München, Germany

**Keywords:** Obesity, Risk factors

## Abstract

**Background/objectives:**

The association between vitamin D and DSPN has been investigated in cross-sectional studies in individuals with diabetes. However, evidence from prospective and population-based studies is still lacking. Also, the potential modifying effect of obesity and glucose tolerance has not been investigated. Therefore, we examined the cross-sectional and prospective associations of serum 25(OH)D with DSPN and assessed possible effect modifications.

**Subjects/methods:**

The study included individuals aged 62–81 years who participated in the German KORA F4 (2006–2008) and FF4 (2013–2014) studies. DSPN was assessed using the Michigan Neuropathy Screening Instrument. Cross-sectional analyses (*n* = 1065; 33% of the participants had obesity) assessed the associations of baseline 25(OH)D with prevalent DSPN, while prospective analyses (*n* = 422) assessed the associations of 25(OH)D with incident DSPN.

**Results:**

No association was found between 25(OH)D and prevalent DSPN in the total sample after adjustment for age, sex, season of blood sampling, BMI, metabolic variables, lifestyle factors, and comorbidities. However, a decrease by 10 nmol/L in 25(OH)D was associated with prevalent DSPN (RR (95% CI) 1.08 (1.01, 1.16)) in individuals with obesity but not in normal-weight individuals (RR (95% CI) 0.97 (0.92, 1.02), *p*_interaction_ = 0.002). No evidence for effect modification by glucose tolerance was found (*p* > 0.05). In the prospective analysis, 25(OH)D levels in the first and second tertiles were associated with higher risk of DSPN (RR (95% CI) 1.18 (1.02; 1.38) and 1.40 (1.04; 1.90)) compared to the third tertile after adjustment for age, sex, season of blood sampling, and BMI. There was no evidence for effect modification by obesity or glucose tolerance categories.

**Conclusions:**

Our study did not show consistent evidence for cross-sectional and prospective associations between serum 25(OH)D levels and DSPN in the total study population of older individuals. However, there was evidence for an association between lower serum 25(OH)D levels and higher prevalence of DSPN in individuals with obesity.

## Introduction

Distal sensorimotor polyneuropathy (DSPN) is often considered a late complication of diabetes with a prevalence of up to 50% in people with longer disease duration [1]. However, increasing evidence suggests that DSPN is also more prevalent in people with prediabetes compared to those with normal glucose tolerance [2, 3] and in individuals with obesity compared to normal-weight individuals [4, 5]. Given the high burden of DSPN [6] and the lack of effective therapeutic options, other modifiable risk factors should also be investigated.

Among these modifiable risk factors, vitamin D is determined by exposure to sunlight, dietary sources or supplementation [7]. Increasing evidence shows associations between low vitamin D levels, as assessed by serum 25-hydroxyvitamin D (25(OH)D), and diseases of the central nervous system [8]. In vitro and in vivo studies indicated that vitamin D is also an essential player in the peripheral nervous system [9] that regulates neural development and function by promoting neuronal cell differentiation [10], enhancing neurotrophin expression [11, 12] and improving myelinisation after injury [13]. Based on these preclinical findings, and given the link between low vitamin D levels and diabetes, particularly in older individuals [14], low vitamin D has been singled out as a potential risk factor in the pathogenesis of DSPN.

Epidemiological studies in individuals with diabetes showed an association of low vitamin D levels with both signs and symptoms of DSPN [15–21], impaired nerve conduction velocity [18, 19, 21–25] and neuropathic pain [19, 26]. A meta-analysis including 1,048 Caucasian individuals with diabetes reported an association between vitamin D deficiency and prevalence of DSPN [27]. These findings were confirmed in Asian individuals with diabetes [28]. Moreover, two recent meta-analyses also reported a link between poor vitamin D status and various lower extremity complications such as diabetic foot ulcers [29] and diabetic foot infection [30].

As the association between vitamin D and DSPN has mostly been investigated in cross-sectional studies and only in individuals with diabetes, it is yet unknown whether there is a temporal relationship between vitamin D and DSPN and whether such an association also exists in individuals with prediabetes. Also, most previous studies were small and did not adjust for the season of blood sampling, an important determinant of vitamin D levels. Moreover, despite the established causal relationship between obesity and low vitamin D status [31] and evidence indicating a positive association between obesity and DSPN [4], no previous study examined effect modification by obesity. Given these limitations, evidence from large-scale, well-designed prospective studies taking into account obesity status in older individuals with different glucose tolerance categories is required.

Therefore, this study aimed to examine cross-sectional and prospective associations between circulating levels of 25(OH)D and DSPN and explore a possible modifying effect of obesity and glucose tolerance categories on these associations in a large population-based cohort of individuals aged 62–81 years from Germany.

## Material and methods

### Study design and study population

This study was based on data from the Cooperative Health Research in the Region of Augsburg (KORA) F4 study (2006–2008) and KORA FF4 study (2013–2014), both follow-up examinations of the population-based KORA S4 study (1999–2001) conducted in Augsburg and two adjacent counties in Southern Germany. The design of the KORA study has been described elsewhere [32]. The study was carried out in accordance with the Declaration of Helsinki, including obtaining written informed consent from all participants and approved by the ethics board of the Bavarian Chamber of Physicians (Munich, Germany).

**Fig. 1:**
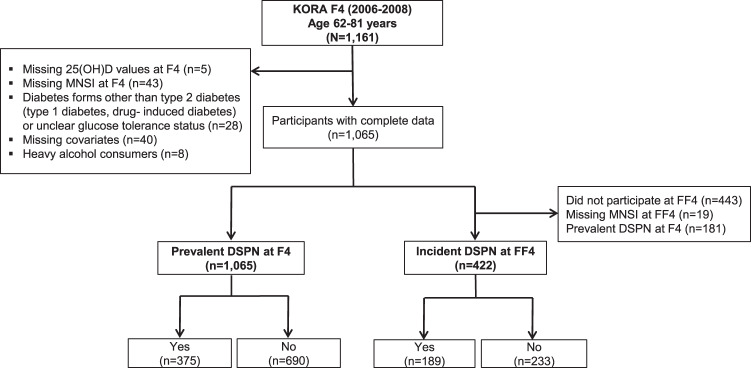
Flow chart describing the selection of the study population. 25(OH)D, 25-hydroxyvitamin D; DSPN, distal sensorimotor polyneuropathy; KORA, Cooperative Health Research in the Augsburg Region; MNSI, Michigan Neuropathy Screening Instrument.

We included 1,161 participants of the KORA F4 study aged 62–81 years (i.e., the age group for whom DSPN was assessed). As described in Fig. 1, exclusions for various reasons resulted in a sample size of 1065 participants for the complete case analysis of prevalent DSPN. For the prospective analysis, we further excluded 443 participants who did not participate at FF4 for various reasons (i.e., illness, unavailability, non-interest or change of the geographic location), 19 participants with missing data for the Michigan Neuropathy Screening Instrument (MNSI) score at FF4, and 181 participants with prevalent DSPN at F4. These exclusions resulted in a sample size of 422 participants for the complete case analysis of incident DSPN.

### Assessment of glucose tolerance categories

After an overnight fast, all participants without known diabetes in KORA F4/FF4 received a standard 75-g oral glucose tolerance test (OGTT). Four glucose tolerance categories were defined using fasting and 2-h glucose levels according to the American Diabetes Association criteria: normal glucose tolerance (NGT), impaired fasting glucose (IFG), impaired glucose tolerance (IGT), and type 2 diabetes (T2D). IFG and/or IGT were considered as “prediabetes”. Known T2D in KORA F4 was defined as a self-report that was validated by the responsible physician or as the current self-reported use of glucose-lowering drugs.

### Measurement of serum 25(OH)D

At F4, serum 25-hydroxyvitamin D (25(OH)D) concentrations were measured with the LIAISON® 25OH Vitamin D TOTAL Assay (DiaSorin Inc., Stillwater, MN, USA). The minimum detectable limit was 10 nmol/L, the interassay coefficient of variation (CV) was 8.7% and 9.1% for target values of 36.75 nmol/L and 118.75 nmol/L, respectively.

### Assessment of DSPN

DSPN was assessed using the foot examination part of the MNSI, which includes four items: the appearance of feet (0 = normal, 1 = abnormal), foot ulcers (0 = normal, 1 = abnormal), ankle reflexes (0 = present, 0.5 = present with reinforcement, 1 = absent) and the vibration perception at the great toes (0 = present, 0.5 = reduced, 1 = absent) [33]. Age-dependent limits were used to classify the normal vibration perception threshold [34]. MNSI was extended by bilateral examination of sensory perception using a 10-g monofilament (Neuropen) (0 = normal, 1 = abnormal), resulting in a total MNSI score ranging from 0 (all aspects are normal) to a maximum of 10 points. An MNSI score >2 points was used to define prevalent and incident DSPN as previously suggested [32, 35]. This definition satisfies the diagnostic criteria for “possible DSPN” according to the Toronto Diabetic Neuropathy Expert Group [36].

### Assessment of covariates

At the F4 visit, body weight and height were measured using standardized equipment and procedures. Body mass index (BMI) was calculated as weight (in kilograms) divided by height (in meters squared). Waist circumference (WC) was measured midway between the lower rib margin and the iliac crest. Obesity (BMI ≥ 30 kg/m^2^) and abdominal obesity (WC ≥ 102 cm for men and ≥88 cm for women) were defined following the NIH cutoff points [37].

For each participant, morning blood was collected and serum samples were aliquoted and stored until analysis. The date of blood draw was used to determine the season for 25(OH)D measurement: fall (September–November), winter (December–February), spring (March–May) and summer (June–August). HbA1c was measured using cation-exchange high-performance liquid chromatographic photometric assays on an Adams HA-8160 hemoglobin analysis system (Menarini Diagnostics, Florence, Italy) [38]. Total cholesterol and triacylglycerol were measured using a Dimension RxL instrument (Dade Behring) [38]. The estimated glomerular filtration rate (eGFR, mL/min/1.73m^2^) was calculated according to the Chronic Kidney Disease Epidemiology Collaboration (CKD-EPI) equation (2012) based on both serum creatinine and serum cystatin C [39]. Beta-nerve growth factor (beta-NGF) was measured with the inflammation panel from OLINK proteomics (Uppsala, Sweden) as described before [40].

Information on age, lifestyle and medical history was obtained during the F4 visit by trained interviewers using standardized questionnaires. Alcohol consumption was graded as none (0 g/day), moderate (<20 g/day for women, <40 g/day for men), high (20–<60 g/day for women, 40–<80 g/day for men) and heavy (≥60 g/day for women, ≥80 g/day for men). Smoking status was categorized into current smokers (including regular and irregular smokers), former smokers and never smokers. Physical activity was assessed according to duration of leisure time activities ([1] >2 h/week, [2] 1–2 h/week, [3] <1 h/week, [4] none) separately in winter and in summer. Participants who had a total sum score for physical activity in winter and in summer ≥5 were classifed as “physically inactive”, otherwise “physically active”. Hypertension was defined as systolic blood pressure ≥140 mmHg and/or diastolic blood pressure ≥90 mmHg and/or use of anti-hypertensive medication given that participants were aware of having hypertension. Self-reported history of stroke and myocardial infarction (yes/no) were also recorded.

Information on the regular use of lipid-lowering drugs and non-steroidal anti-inflammatory drugs (NSAIDs) during the last 7 days was collected through a database-supported computer software as described in detail elsewhere [32]. Briefly, participants were asked to bring all medications to the study center for verification of use. Intake of vitamin D supplements or cod liver oil during the last 6 weeks was assessed by a separate interview question.

### Statistical analysis

Summary results are presented as medians (25th percentile, 75th percentile) or percentages. Differences in characteristics between participants with and without DSPN were calculated with the Kruskal–Wallis non-parametric rank test and the chi-square test. Correlations of serum 25(OH)D with continuous variables were estimated with Spearman’s correlation coefficients.

The associations of baseline serum 25(OH)D with prevalent and incident DSPN (as main dependent variables) were assessed with Poisson regression with robust error variance. Four models with increasing complexity were built to assess the impact of the covariates. Model 1 (minimally adjusted) included sex, age and season of blood draw as covariates. Model 2 was additionally adjusted for BMI. Model 3 was further adjusted for HbA1c, total cholesterol, log-transformed triacylglycerol, physical activity, alcohol consumption, smoking, eGFR, hypertension, stroke, myocardial infarction, use of lipid-lowering drugs and use of non-steroidal anti-inflammatory drugs. Lastly, we adjusted for vitamin D supplementation in model 4 (fully adjusted main model). All covariates used in the four regression models were measured at baseline (F4). Results are presented as risk ratios (RRs) with their 95% confidence intervals (CIs). Baseline serum 25(OH)D levels were modeled as a continuous variable (per 10 nmol/L decrease) and as a categorical variable categorized into tertiles. In the analysis using tertiles, tertile three was used as the reference category, and the linear trend test was performed by treating serum 25(OH)D as a continuous variable.

To explore the potential effect modification of obesity and glucose tolerance categories (T2D vs. prediabetes/NGT or NGT vs. prediabetes/T2D) on the associations between baseline serum 25(OH)D and prevalent and incident DSPN, we performed stratified analyses in the fully adjusted model (model 4). *p*-values for interaction were obtained by adding a cross-product term between 25(OH)D and the potential effect modifier.

We used restricted cubic splines to assess the shape of the association between 25(OH)D and the MNSI score (continuous outcome) [41]. Knots were placed at the 5th, 50th, and 95th percentiles of baseline serum 25(OH)D.

All statistical tests were 2-sided with an alpha level of 0.05, and all analyses were carried out using SAS statistical software (version 9.4; SAS Institute, Inc., Cary, NC, USA).

## Results

### Characteristics of the study population

Baseline characteristics of the 1065 participants included in the cross-sectional analysis are presented in Table 1. The median age was 70 years, and 51% were men. Obesity was present in 33% of the study sample, abdominal obesity in 60%, T2D in 22% and serum 25(OH)D levels <25 nmol/L in 17%. The median baseline serum 25(OH)D level was 44.3 nmol/L. DSPN was present in 35% of the study sample. Compared to individuals without prevalent DSPN, those with prevalent DSPN were older, taller and more likely to have obesity and abdominal obesity and higher HbA1c values. However, they had lower cholesterol levels and lower eGFR values. In addition, they reported higher alcohol consumption, less physical activity and had a higher prevalence of T2D and myocardial infarction.

**Table 1:** Baseline characteristics of the study population used in the analysis of prevalent DSPN.

Baseline characteristics	Sample for the prevalence analysis (*n* = 1065)	Prevalent DSPN at F4		
		Yes (*n* = 375, 35%)	No (*n* = 690, 65%)	*p*
Age (years)	70 (66; 74)	72 (67; 77)	69 (65; 73)	**<0.0001**
Sex (male, %)	51%	55%	49%	0.063
Height (cm)	165 (159; 172)	167 (160; 173)	165 (158; 172)	**0.0063**
BMI (kg/m^2^)	28.3 (25.6; 31.2)	29.0 (26.5; 32.4)	27.7 (25.3; 30.7)	**<0.0001**
Obesity (%)^a^	33%	40%	30%	**0.002**
Waist circumference (cm)	98.0 (90.1; 105.8)	101.3 (93.8; 109.8)	96.0 (89.0; 104.1)	**<0.0001**
Abdominal obesity (%)^b^	60%	68%	56%	**0.0001**
Total cholesterol (mmol/L)	5.7 (5.0; 6.4)	5.5 (4.8; 6.2)	5.7 (5.1; 6.4)	**<0.0001**
Triacylglycerol (mmol/L)	1.3 (1.0; 1.8)	1.3 (0.9; 1.8)	1.3 (1.0; 1.8)	0.515
HbA1c (%)	5.7 (5.4; 5.9)	5.7 (5.4; 6.1)	5.6 (5.4; 5.9)	**0.0004**
HbA1c (mmol/mol)	39 (36; 41)	39 (36; 43)	38 (36; 41)	**0.0004**
eGFR (mL/min/1.73m^2^)	77.5 (67.4; 87.5)	74.0 (62.6; 84.5)	79.4 (70.2; 89.1)	**<0.0001**
Smoking status (%)				0.057
Current smoker	7%	5%	9%	
Former smoker	7%	42%	42%	
Never smoker	42%	54%	49%	
Alcohol consumption (%)^c^				**0.008**
None	32%	37%	30%	
Moderate	58%	51%	61%	
High	10%	12%	9%	
Physically active (%)^d^	50%	41%	56%	**<0.0001**
Glucose tolerance status (%)				**<0.0001**
NGT	39%	32%	42%	
IFG and/or IGT	40%	39%	41%	
Type 2 diabetes	22%	29%	17%	
Stroke (%)	4%	5%	3%	0.085
Myocardial infarction (%)	6%	8%	5%	**0.039**
Hypertension (%)	62%	64%	61%	0.361
Lipid-lowering drugs (%)	25%	26%	25%	0.628
NSAIDs (%)	4%	6%	3%	0.056
Vitamin D supplementation (%)	6%	5%	7%	0.185
Season of blood draw (%)				**0.033**
Fall	28%	27%	28%	
Winter	31%	27%	34%	
Spring	25%	27%	25%	
Summer	15%	19%	13%	
Serum 25(OH)D (nmol/L)	44.3 (30.7; 61.2)	43.1 (28.1; 60.3)	44.7 (31.2; 61.5)	0.150
Serum 25(OH)D categories (%)				0.257
<25 nmol/L	17%	19%	16%	
≥25–<50 nmol/L	43%	43%	43%	
≥50 nmol/L	40%	38%	42%	
Beta-NGF(NPX)	1.84 (1.67; 2.01)	1.89 (1.70; 2.08)	1.81 (1.66; 1.99)	**<0.0001**

Summary results are presented as percentages or median (25th percentile; 75th percentile).

Beta-NGF has 38 missing values.

*25(OH)D* 25-hydroxyvitamin D, *beta-NGF* beta-nerve growth factor, *BMI* body mass index, *eGFR* estimated glomerular filtration rate, *IFG* impaired fasting glucose, *IGT* impaired glucose tolerance, *MNSI* Michigan neuropathy screening instrument, *NGT* normal glucose tolerance, *NPX* normalized protein expression, *NSAIDs* non-steroidal anti-inflammatory drugs.

^a^Obesity was defined as BMI ≥ 30 kg/m^2^.

^b^Abdominal obesity was defined as waist circumference ≥102 cm for men and ≥88 cm for women.

^c^Alcohol consumption was graded as none (0 g/day), moderate (<20 g/day for women and <40 g/day for men), high (20–<60 g/day for women and 40–<80 g/day for men), and heavy (≥60 g/day for women and ≥80 g/day for men).

^d^Physical activity was assessed according to the duration of leisure time activities ((1) >2 h/week, (2) 1–2 h/week, (3) <1 h/week, (4) none) separately in winter and in summer. Participants with a total sum score for physical activity in winter and in summer of 5 or more were classified as “physically inactive”, otherwise “physically active”.

Bold values identify statistical significance (*p* < 0.05).

Baseline characteristics of the 422 participants included in the prospective analysis are presented in Table 2. Participants were followed for a mean duration of 6.5 (standard deviation 0.5) years, during which 189 (45%) developed DSPN. Individuals with incident DSPN were older, taller and had higher BMI and waist circumference, more often hypertension but lower eGFR values. They were also more likely to be physically inactive.

**Table 2:** Baseline characteristics of the study population used in the analysis of incident DSPN.

Baseline characteristics	Sample for the incidence analysis (*n* = 422)	Incident DSPN at FF4		
		Yes (*n* = 189, 45%)	No (*n* = 233, 55%)	*p*
Age (years)	67 (64; 71)	68 (65; 72)	67 (64; 70)	**0.0008**
Sex; male (%)	50%	55%	47%	0.092
Height (cm)	166 (159; 172)	168 (160; 174)	165 (159; 171)	**0.032**
BMI (kg/m^2^)	27.5 (25.3; 30.3)	27.7 (25.8; 30.7)	27.0 (24.7; 30.0)	**0.014**
Obesity (%)^a^	27%	31%	24%	0.122
Waist circumference (cm)	95.2 (88.4; 103.3)	97.7 (90.6; 105.4)	93.1 (85.7; 101.0)	**<0.0001**
Abdominal obesity (%)^b^	51%	61%	43%	**0.0003**
Total cholesterol (mmol/L)	5.8 (5.1; 6.4)	5.7 (4.9; 6.4)	5.8 (5.2; 6.5)	0.166
Triacylglycerol (mmol/L)	1.3 (1.0; 1.8)	1.3 (1.0; 1.9)	1.3 (1.0; 1.7)	0.415
HbA1c (%)	5.6 (5.4; 5.9)	5.7 (5.4; 5.9)	5.6 (5.4; 5.8)	0.137
HbA1c (mmol/mol)	38 (36; 41)	39 (36; 41)	38 (36; 40)	0.137
eGFR (mL/min/1.73m^2^)	81.0 (72.9; 90.0)	79.5 (72.0; 89.1)	82.0 (74.1; 91.0)	**0.032**
Smoking status (%)				0.827
Current smoker	7%	8%	7%	
Former smoker	42%	41%	42%	
Never smoker	51%	50%	51%	
Alcohol consumption (%)^c^				0.756
None	30%	31%	29%	
Moderate	59%	59%	59%	
High	11%	10%	12%	
Physically active (%)^d^	59%	53%	64%	**0.028**
Glucose tolerance status (%)				0.630
NGT	45%	43%	46%	
IFG and/or IGT	40%	42%	38%	
Type 2 diabetes	15%	14%	16%	
Stroke (%)	1%	2%	1%	0.222
Myocardial infarction (%)	5%	6%	4%	0.473
Hypertension (%)	59%	66%	53%	**0.007**
Lipid-lowering drugs (%)	25%	25%	26%	0.836
NSAIDs (%)	1%	1%	1%	0.829
Vitamin D supplementation (%)	7%	7%	7%	0.830
Season of blood draw (%)				0.385
Fall	26%	29%	24%	
Winter	38%	34%	41%	
Spring	20%	20%	21%	
Summer	15%	17%	14%	
Serum 25(OH)D (nmol/L)	46.8 (33.1; 64.3)	46.7 (31.9; 64.3)	47.0 (34.9; 63.0)	0.449
Serum 25(OH)D categories (%)				0.443
<25 nmol/L	13%	15%	11%	
≥25–<50 nmol/L	43%	42%	43%	
≥50 nmol/L	44%	43%	46%	
Beta-NGF (NPX)	1.80 (1.63; 1.96)	1.84 (1.69; 2.01)	1.78 (1.57; 1.91)	**0.0018**

Summary results are presented as percentages or median (25th percentile; 75th percentile).

Beta-NGF has 13 missing values.

*25(OH)D* 25-hydroxyvitamin D, *beta-NGF* beta-nerve growth factor, *BMI* body mass index, *eGFR* estimated glomerular filtration rate, *IFG* impaired fasting glucose, *IGT* impaired glucose tolerance, *MNSI* Michigan neuropathy screening instrument, *NGT* normal glucose tolerance, *NPX* Normalized Protein eXpression, *NSAIDs* non-steroidal anti-inflammatory drugs.

^a^Obesity was defined as BMI ≥ 30 kg/m^2^.

^b^Abdominal obesity was defined as waist circumference ≥102 cm for men and ≥88 cm for women.

^c^Alcohol consumption was graded as none (0 g/day), moderate (<20 g/day for women and <40 g/day for men), high (20–<60 g/day for women and 40–<80 g/day for men), and heavy (≥60 g/day for women and ≥80 g/day for men).

^d^Physical activity was assessed according to the duration of leisure time activities ((1) >2 h/week, (2) 1–2 h/week, (3) <1 h/week, (4) none) separately in winter and in summer. Participants with a total sum score for physical activity in winter and in summer of 5 or more were classified “physically inactive”, otherwise “physically active”.

Bold values identify statistical significance (*p* < 0.05).

Spearman correlation showed that older age, higher BMI, poor glycaemic control, higher cholesterol, and triacylglycerol levels were all negatively correlated with serum 25(OH)D (all *p* < 0.05). Higher eGFR values were positively correlated with serum 25(OH)D (*p* = 0.005). There was no correlation between 25(OH)D and beta-NGF (*p* = 0.96).

### Association of serum 25(OH)D with prevalent DSPN

In the total sample, there was no significant association between serum 25(OH)D and prevalent DSPN in all models using 25(OH)D as a continuous variable or tertiles (Table 3). However, in the fully adjusted model, the analysis stratified by obesity (Table 4) identified a significant association between a lower serum 25(OH)D levels and prevalent DSPN in participants with obesity (RR (95% CI) per 10 nmol/L; 1.08 (1.01, 1.16)), whereas there was no evidence for an association in participants with normal weight (*p*_interaction_ = 0.002). When 25(OH)D was modeled as tertiles, the association of the lowest vs. the highest tertile was also stronger in participants with obesity (tertile1: RR (95% CI) 1.17 (0.98, 1.39); tertile 2: RR (95% CI) 1.38 (0.97, 1.95) than in normal-weight participants (*p*_interaction_ = 0.028).

**Table 3:** Associations of baseline serum 25(OH)D levels with prevalent and incident DSPN.

		Per 10 nmol/L decrease		25(OH)D (nmol/L) by tertiles^a^			
				T1 (*n* = 355)	T2 (*n* = 357)	T3 (*n* = 353)	
Outcome	Model	RR (95% CI)	*p*	RR (95% CI)	RR (95% CI)		*p*_*trend*_
Prevalent DSPN	Model 1	1.03 (0.98, 1.07)	0.143	1.08 (0.96, 1.21)	1.17 (0.93, 1.47)	Ref.	0.339
	Model 2	1.00 (0.96, 1.04)	0.692	1.04 (0.92, 1.16)	1.08 (0.86, 1.35)	Ref.	0.950
	Model 3	1.00 (0.96, 1.04)	0.843	1.02 (0.91, 1.14)	1.05 (0.83, 1.31)	Ref.	0.811
	Model 4	1.00 (0.96, 1.04)	0.942	1.01 (0.90, 1.13)	1.03 (0.82, 1.29)	Ref.	0.689
Incident DSPN	Model 1	1.02 (0.97, 1.08)	0.274	**1.20 (1.03, 1.40)**	**1.46 (1.08, 1.97)**	Ref.	0.065
	Model 2	1.02 (0.96, 1.07)	0.437	**1.18 (1.02, 1.38)**	**1.40 (1.04, 1.90)**	Ref.	0.126
	Model 3	1.01 (0.95, 1.07)	0.716	1.14 (0.97, 1.35)	1.32 (0.95, 1.82)	Ref.	0.247
	Model 4	1.01 (0.95, 1.07)	0.657	1.15 (0.98, 1.35)	1.33 (0.96, 1.84)	Ref.	0.217

Model 1: adjusted for sex, age (continuous) and season of blood draw.

Model 2: adjusted for model 1+ BMI (continuous).

Model 3: adjusted for model 2 + HbA1c (continuous), total cholesterol (continuous), triacylglycerol (log-transformed, continuous), physical activity (active/inactive), alcohol consumption (none/moderate/high), smoking (current/former/never), eGFR (continuous), hypertension (yes/no), stroke (yes/no), myocardial infarction (yes/no), use of lipid-lowering drugs (yes/no) and use of non-steroidal anti-inflammatory drugs (yes/no).

Model 4: adjusted for model 3+ vitamin D supplementation (yes/no).

*25(OH)D* 25-hydroxyvitamin D, *CI* confidence interval, *DSPN* distal sensorimotor polyneuropathy, *RR* risk ratio, *Ref*. reference, *T* tertile.

^a^Tertile cutoff points: 35.47, 55.17 nmol/L.

Bold values identify statistical significance (*p* < 0.05).

**Table 4:** Associations of baseline serum 25(OH)D levels with prevalent and incident DSPN according to obesity.

			Per 10 nmol/L decrease		25(OH)D (nmol/L) by tertiles*			
					T1	T2	T3	
Outcome		*n*	RR (95% CI)	*p*_interaction_	RR (95% CI)	RR (95% CI)		*p*_interaction_
Prevalent DSPN	Individuals with obesity	357	**1.08 (1.01,** **1.16)**	**0.002**	1.17 (0.98, 1.39)	1.38 (0.97, 1.95)	Ref.	**0.028**
	Individuals with normal weight	708	0.97 (0.92, 1.02)		0.92 (0.78, 1.09)	0.85 (0.61, 1.19)	Ref.	
Incident DSPN	Individuals with obesity	116	1.09 (0.91, 1.30)	0.269	1.22 (0.77, 1.92)	1.49 (0.60, 3.70)	Ref.	0.209
	Individuals with normal weight	306	1.00 (0.93, 1.06)		1.19 (0.97, 1.47)	1.43 (0.95, 2.16)	Ref.	

Associations adjusted for sex, age (continuous), season of blood draw, HbA1c (continuous), total cholesterol (continuous), triacylglycerol (log-transformed, continuous), physical activity (active/inactive), alcohol consumption (none/moderate/high), smoking (current/former/never), eGFR (continuous), hypertension (yes/no), stroke (yes/no), myocardial infarction (yes/no), use of lipid-lowering drugs (yes/no), use of non-steroidal anti-inflammatory drugs (yes/no), vitamin D supplementation (yes/no).

Obesity is defined as BMI ≥ 30 kg/m^2^.

*25(OH)D* 25-hydroxyvitamin D, *CI* confidence interval, *DSPN* distal sensorimotor polyneuropathy, *RR* risk ratio, *Ref*., reference, *T* tertile.

^a^Tertile cutoff points: 35.47, 55.17 nmol/L.

Bold values identify statistical significance (*p* < 0.05).

**Fig. 2:**
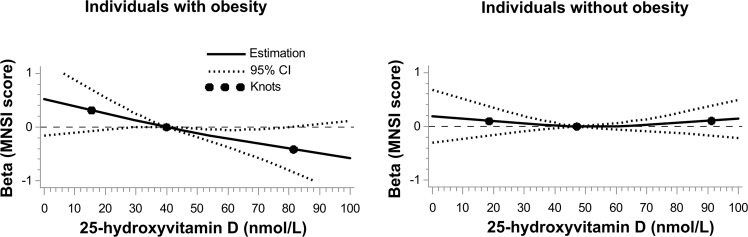
Dose–response relationship plots of the association between serum 25-hydroxyvitamin D levels and MNSI score at F4 in individuals with obesity (left panel) and individuals without obesity (right panel). Black lines, estimate; dashed lines, confidence limits; dots, knots set at the 5th, 50th, and 95th percentile of serum 25-hydroxyvitamin D. The reference was the 50th percentile. Associations were adjusted for sex, age (continuous), season of blood draw, HbA1c (continuous), total cholesterol (continuous), triacylglycerol (log-transformed, continuous), physical activity (active/inactive), alcohol consumption (none/moderate/high), smoking (current/former/never), eGFR (continuous), hypertension (yes/no), stroke (yes/no), myocardial infarction (yes/no), use of lipid-lowering drugs (yes/no), use of non-steroidal anti-inflammatory drugs (yes/no), and vitamin D supplementation (yes/no).

To assess the shape of the association between 25(OH)D and DSPN, we incorporated restricted cubic splines into a multivariable regression model with MNSI score at F4 as an outcome in individuals with obesity and normal-weight individuals separately (Fig. 2). In participants with obesity, the association between serum 25(OH)D and MNSI score was linear (*p* for nonlinearity = 0.790; *p* for overall association = 0.022), whereas in the normal-weight participants, the association was also linear (*p* for nonlinearity = 0.373) but not significant (*p* for overall association = 0.663).

There was no interaction between serum 25(OH)D and glucose tolerance categories (*p*_interaction_ = 0.939 for NGT vs. prediabetes/T2D, *p*_interaction_ = 0.279 for NGT/prediabetes vs. T2D).

### Association between 25(OH)D and incident DSPN

Table 3 shows the associations between serum 25(OH)D and incident DSPN in the total sample (*n* = 422). No significant association was found between 25(OH)D as a continuous variable and incident DSPN. However, in model 1 adjusted for age, sex and season, serum 25(OH)D levels in the first and second tertiles were associated with a higher risk of incident DSPN (RR (95% CI) 1.20 (1.03, 1.40) and RR (95% CI) 1.46 (1.08, 1.97), respectively) compared to tertile 3. These associations remained robust after additional adjustment for BMI but were attenuated after full adjustment for covariates.

Stratified analysis by obesity did not show any difference between participants with obesity and those with normal weight (*p*_interaction_ = 0.269 for 10 nmol/L and *p*_interaction_ = 0.209 for tertiles).

There was also no interaction between serum 25(OH)D and glucose tolerance categories (*p*_interaction_ = 0.094 for NGT vs. prediabetes/T2D, *p*_interaction_ = 0.722 for NGT/prediabetes vs. T2D).

## Discussion

This study showed that lower serum 25(OH)D was associated with prevalent DSPN in individuals with obesity but not in normal-weight individuals in the older population. There were no differences in the associations according to glucose tolerance categories. Prospective results showed an association between lower serum 25(OH)D and higher risk of DSPN in the total sample, which was abolished after extensive adjustment for confounders.

Previous epidemiological studies reported an association between low serum 25(OH)D and DSPN [15–26]. However, all these studies were predominantly conducted in individuals with type 2 diabetes, and none examined the association between serum 25(OH)D and DSPN in individuals with and without obesity. Our study is the first to use a population-based design and to assess effect modification by obesity. Studies examining the association between 25(OH)D and DSPN only in individuals with diabetes might have limitations. First, they do not consider recent research indicating the increasing prevalence of DSPN in prediabetes [2, 42]. Second, observational studies [14], Mendelian randomization studies [43] and several RCTs [44] suggest a role of low 25(OH)D levels in increasing insulin resistance, decreasing β-cell function and promoting the development of T2D. Thus, even after adjusting for diabetes duration and glycaemic control, it is difficult to know whether the observed associations between low 25(OH)D levels and DSPN are genuine associations or rather an epiphenomenon as previously suggested [18].

Obesity has long been associated with low vitamin D status irrespective of age and ethnicity [45]. Using a genetic approach that limits confounding, a Mendelian randomization study including 21 cohorts and up to 42,000 participants confirmed a causal association between higher BMI and lower circulating 25(OH)D levels [31]. Several mechanisms were suggested to explain lower vitamin D in individuals with obesity. Formerly, vitamin D was thought to sequestrate in the excessive adipose tissue in individuals with obesity, decreasing its release in the circulation [45]. Recent studies suggested that a volumetric dilution rather than sequestration best explains the low vitamin D status in individuals with obesity [46] and that obesity decreases the activity of the hepatic 25-hydroxylase enzyme responsible for converting vitamin D into 25(OH)D [47].

Of note, obesity increases the risk of DSPN both in older individuals from the general population [4] and in those with type 2 diabetes [5]. Obesity is associated with chronic systemic inflammation where enlarged adipocytes release a variety of pro-inflammatory adipokines [48]. Using data from the KORA study and a mediation analysis approach, inflammation has been proposed as a biologically plausible link between obesity and DSPN [4]. In particular, three biomarkers of subclinical inflammation, including two chemokines (C-C motif chemokine ligand 7 [CCL7], C-X-C motif chemokine ligand 10 [CXCL10]) and the soluble form of the transmembrane receptor (Delta/Notch-like epidermal growth factor-related receptor [DNER]) partially mediate the association between obesity and DSPN [4].

Our data did not show a significant link between serum 25(OH)D and DSPN in the total sample in the cross-sectional and prospective analyses. Moreover, we believe that the magnitude of the associations in individuals with obesity is small. Nevertheless, given previous epidemiologic evidence linking vitamin D to DSPN, we cannot rule out a possible role of vitamin D in DSPN.

Several mechanisms may explain the role of vitamin D in the pathogenesis of DSPN. First, vitamin D has neuroprotective actions, which might be reduced in low 25(OH)D status. In vitro studies using murine [12] and rat cells [49] showed that treatment of Schwann cells with 1,25(OH)2D3, the active form of vitamin D, stimulates NGF and insulin-like growth factor (IGF-1) expression. NGF is a neurotrophin that plays an essential role in repairing nerve damage [50]. IGF-1 is a growth factor that stimulates neurogenesis and accelerates myelination and axonal regrowth [11]. We observed higher levels of beta-NGF in individuals with prevalent and incident DSPN, which might be explained by an upregulation to promote nerve regeneration and repair. However, no correlation between 25(OH)D and beta-NGF was found in our study, in contrast to a study in individuals with type 1 diabetes, which reported a positive correlation [17]. A study in rodent sciatic nerves showed that treatment with vitamin D protects Schwann cells against apoptosis induced by advanced glycation end-products [51]. In vitro studies using human cells demonstrated that treating neuroblastoma cells with vitamin D and other growth factors induced neuronal cell differentiation and generated sustainable and well-differentiated neurons [10]. Furthermore, studies in vitamin D receptor-deficient mice underlined the role of vitamin D in axonal homogeneity [49].

Second, vitamin D has anti-inflammatory and immunomodulatory actions, which might be reduced in low 25(OH)D status contributing to the pathogenesis of DSPN. Notably, a severe vitamin D deficiency has been found associated with increased expression of inflammatory cytokines [52], particularly interleukin-6 and tumor necrosis factor-α, both previously reported to increase the risk of DSPN [53].

In individuals with obesity and low vitamin D, higher circulating levels of biomarkers of inflammation due to obesity along with reduced anti-inflammatory and neuroprotective actions of vitamin D could represent mechanisms that might explain the association between low 25(OH)D levels and prevalent DSPN in our study.

Studies comparing serum 25(OH)D in individuals with diabetes showed lower serum or plasma 25(OH)D levels in individuals with DSPN than those without DSPN [15, 16, 21, 22, 24, 25] in contrast to our results. However, we still did find an association between low 25(OH)D levels and incident DSPN in the initial models. Differences in findings might be explained by the study population (individuals with diabetes vs. individuals from the general population), by between-study heterogeneity in the assessment of DSPN (i.e., self-report, neuropathy symptom score, neuropathy disability score, vibration perception threshold, Semmes-Weinstein monofilament testing and nerve conduction studies) and by the more extensive adjustment for potential confounders in our study. In particular, it is important to note that our study is the only study that took into account the confounding effect of the season on the associations between 25(OH)D and DSPN. Despite the known seasonal fluctuation of vitamin D levels, none of the previous studies had adjusted for the season of blood draw for 25(OH)D measurements except one study, which adjusted for sunlight exposure and daily activity. However, it included only individuals for whom blood was collected during the summer [26].

Our study shows that only 6% of the participants took vitamin D supplements or cod liver oil. This proportion was below our expectations given the widespread use of vitamin D supplements among older individuals. An explanation could be the neglect of vitamin D supplementation in older individuals, often having a high proportion of polypharmacy to manage ageing-related diseases. Intervention studies testing the effect of vitamin D supplementation on neuropathic pain indicated that supplementation with high, single or weekly dosages of vitamin D was associated with a significant decrease in the signs and symptoms of DSPN [54, 55]. However, these trials were small-scale [56], not placebo-controlled, restricted to individuals with T2D and painful diabetic neuropathy, and supplemented with vitamin D doses based on a “one-size-fits-all” approach without considering individual differences. However, it remains unknown whether vitamin D supplementation could be an effective therapeutic regimen that can change the pathological features of DSPN and its progression. It is worth noting that vitamin D supplementation in other neurological diseases did not seem effective in modifying the disease phenotype for reasons probably related to patient selection. Therefore, future trials assessing the efficacy of vitamin D supplementation as a disease-modifying treatment for DSPN should carefully consider individual differences, particularly differences in obesity status, to increase their ability to detect the supplementation’s beneficial effect.

### Strengths and limitations

This study includes a large sample size, and its design allows for both cross-sectional and prospective analyses with extended follow-up and comprehensive adjustment for confounders. Also, KORA is a population-based study, which is optimal to test the hypothesis of effect modifications by obesity or glucose tolerance categories on the associations between 25(OH)D and DSPN. Some limitations of our study should be noted. First, the inclusion of white and older individuals from one country limits the generalizability of our findings. As vitamin D is mainly synthesized in the skin and strongly depends on skin color and age, future studies should aim to replicate our findings in populations of other ethnicities and among young and middle-aged individuals. Another limitation of this study is that 25(OH)D was not measured with gold standard methods, i.e., high-performance liquid chromatography–electrospray ionization–mass spectroscopy. DSPN was not assessed with nerve conduction studies, thus does not satisfy the definition of “confirmed DSPN”. A further limitation was the smaller sample size of the prospective compared to the cross-sectional analysis. This limitation indicates that our prospective analysis may not have been adequately powered, which may explain the lack of association between serum 25(OH)D levels and incident DSPN in the fully adjusted models. Finally, the observational study design cannot rule out potential residual confounding. Future studies based on Mendelian Randomization (MR) analyses that use instrumental variables (IVs) are needed to strengthen evidence of a causal association between 25(OH)D and DSPN because they are not subject to biases with confounding and reverse causation.

## Conclusion

Our findings from a large, well-designed observational cohort found no consistent associations between serum 25(OH)D and DSPN in cross-sectional and prospective analyses. However, our data suggests an association between lower serum 25(OH)D levels and higher DSPN prevalence in individuals with obesity. Further large-scale prospective population-based studies are required to confirm and extend our findings. In a clinical setting, intervention studies are needed to clarify whether body weight loss (i.e., by bariatric surgery or lifestyle interventions) in individuals with obesity can effectively increase vitamin D levels and subsequently prevent DSPN. Alternatively, well-designed randomized clinical trials are needed to determine the benefits of vitamin D supplementation, taking into account body weight, in preventing the development of DSPN.

## Data Availability

The data are subject to national data protection laws. Therefore, data cannot be made freely available in a public repository. However, data can be requested through an individual project agreement with KORA. To obtain permission to use KORA data under the terms of a project agreement, please use the digital tool KORA.PASST (https://helmholtz-muenchen.managed-otrs.com/external).

## Code availability

The computer code used to generate results of this manuscript can be obtained upon reasonable request from the corresponding author.
